# Pharmacokinetics of Ertugliflozin, a Sodium-Glucose Co-Transporter-2 Inhibitor (SGLT2i) in Horses After Enteral Administration

**DOI:** 10.3390/vetsci13050445

**Published:** 2026-05-01

**Authors:** Naomi C. Kirkwood, Kristopher J. Hughes, Amy L. Lovett, Gregory S. Doran, David I. Rendle, Scott H. Edwards

**Affiliations:** 1 School of Agricultural, Environmental and Veterinary Sciences, Charles Sturt University, Wagga Wagga, NSW 2678, Australia; nkirkwood@csu.edu.au (N.C.K.); krhughes@csu.edu.au (K.J.H.); alovett@csu.edu.au (A.L.L.); gdoran@csu.edu.au (G.S.D.); 2 Gulbali Institute, Charles Sturt University, Albert Pugsley Place, Wagga Wagga, NSW 2678, Australia; 3 EMT Consulting, Tiverton EX16 9JU, UK; daverendle@me.com

**Keywords:** equine metabolic syndrome (EMS), laminitis, equine, horse, pituitary pars intermedia dysfunction (PPID), pharmacodynamics, endocrine, endocrinopathy

## Abstract

Ertugliflozin is a sodium-glucose co-transporter-2 inhibitor that has demonstrated promise as a treatment for hyperinsulinaemia in horses. Despite the frequent use of ertugliflozin in equine clinical practice, the pharmacokinetics of this drug in horses has not been established. The aim of the present study was to determine the pharmacokinetics of one supratherapeutic dose (0.25 mg/kg) of ertugliflozin in eight horses. Horses were defined as being healthy by physical examination, haematological, blood biochemical and oral sugar test (OST) results. Plasma concentrations of ertugliflozin were quantified using high-performance liquid chromatography–tandem mass spectrometry 0, 0.25, 0.5, 0.75, 1, 2, 3, 4, 6, 8, 10, 14, 18, 24, 30, 36, 48, 60, 72, 96, and 120 h after drug administration enterally. Non-compartmental analysis led to determination of key pharmacokinetic variables, including mean ± SD time to maximum concentration (T_max)_ of 0.91 ± 0.13 h, maximum measured concentration (C_max)_ of 267.52 ± 25.37 ng/mL, terminal elimination half-life (T_1/2_) of 17.65 ± 3.15 h and apparent oral clearance (CL/F) of 106.95 ± 27.53 mL/h/kg. No clinical signs of adverse effects or blood biochemical abnormalities occurred after drug administration. The results of this study suggest that a single supratherapeutic dose of ertugliflozin in healthy horses is safe. The pharmacokinetics of enterally administered ertugliflozin in horses are similar to pharmacokinetics of the drug in humans and the long T_1/2_ makes ertugliflozin suitable for once daily dosing in horses. It is proposed that a starting dose for ertugliflozin in horses be in the range 0.05–0.1 mg/kg. Further pharmacokinetic studies are required to optimise the dose regimen for treating horses with hyperinsulinaemia.

## Simple Summary

Ertugliflozin, a sodium-glucose co-transporter-2 inhibitor drug, is used commonly in clinical practice to treat hyperinsulinaemia in horses. However, the pharmacokinetics of ertugliflozin in horses has not been studied, precluding the optimisation of dosing regimens. The aim of the present study was to determine the pharmacokinetic profile of ertugliflozin after enteral administration of a supratherapeutic dose, and to determine the safety of a single supratherapeutic dose in healthy adult horses. The T_max_, C_max_, and T_1/2_ of ertugliflozin was successfully determined, and a recommended dose range was established. Further studies in horses with hyperinsulinaemia are required to consolidate our knowledge of the efficacy of ertugliflozin in horses with hyperinsulinaemia. These contributions will advance professional practice by providing veterinarians with recommended dosing regimens for ertugliflozin and providing advances in the management of hyperinsulinaemia in horses, leading to improvements in horse welfare and health.

## 1. Introduction

Equine metabolic syndrome (EMS) is a collection of risk factors for hyperinsulinaemia-associated laminitis (HAL) and is characterised by insulin dysregulation (ID) [1,2]. Laminitis is a debilitating and life-threatening disease of the equine hoof, and it is well recognised that hyperinsulinaemia can induce laminitis [3]. Caloric restriction, minimisation of dietary non-structural carbohydrates (NSC) and exercise reduce post-prandial hyperinsulinaemia and improve insulin sensitivity in horses [1,4,5]. However, some horses are unable to exercise due to HAL [1], or are refractory to exercise and dietary management, whereby pharmacological treatment can be required to reduce morbidity and improve animal welfare. Metformin, levothyroxine and pioglitazone have been used for the treatment of ID; however, the beneficial effects of these drugs are limited and inconsistent [6,7,8,9,10,11,12,13,14], and the oral bioavailability of metformin is low in horses [7,15].

Glucose reabsorption by sodium-glucose co-transporters (SGLT) is a major contributor to blood glucose concentration and insulin secretion from the β-cells in pancreatic islets [16]. In humans, SGLT-2 and SGLT1 are responsible for 97% and 3% of renal fractional glucose reabsorption, respectively [17,18,19]. In humans with type 2 diabetes mellitus, SGLT-2 inhibitor (SGLT2i) drugs have been used to increase urinary glucose excretion, reduce plasma glucose concentration and subsequently reduce insulin secretion [20,21]. Recently, SGLT2i drugs have been reported to reduce insulin concentrations and improve HAL in horses [22,23,24,25,26,27,28]. Ertugliflozin, a SGLT2i drug, is available as a prescription tablet (Steglatro: Merck Sharpe & Dohme) from human pharmacies in many countries and as a compounded paste or tablet in Australia, the United Kingdom and America (BOVA, Epicur Pharma^®^), and it is used in the management of horses with ID and HAL with promising results [26,27,28]. Despite the use of SGLT2i drugs in veterinary practice [29], the only pharmacokinetic studies of SGLT2i drugs in horses have investigated canagliflozin [30,31] and velagliflozin [32]. However, the results of these studies cannot be extrapolated to other SGLT2i drugs, such as ertugliflozin, as the drugs likely have different dose rates, plasma half-lives, therapeutic plasma concentrations, bioavailability, and SGLT receptor selectivity [33]. The lack of pharmacokinetic studies of ertugliflozin in horses precludes the optimisation of dosing regimens and efficacy of this drug.

The use of SGLT2i drugs is commonly associated with hypertriglyceridaemia in horses [22,23,26,27] and possibly increases the risk of triglyceride-induced hepatic and renal dysfunction [22]. Polyuria and polydipsia (PU/PD) have also been reported in horses that were administered ertugliflozin [26]. Urinary tract infection (UTI) secondary to glucosuria has been suggested as a potential adverse effect of SGLT2i drug use in horses, although no cases have been reported [26]. In humans, meta-analyses investigating UTI risk with SGLT2i drug administration have yielded conflicting results [34,35]. It has been suggested that the risk of UTI secondary to glucosuria might be due to diabetes, [36] or counteracted by concurrent SGLT2i drug-induced osmotic diuresis [36,37], which might also be the case in horses. Hypoglycaemia is another possible adverse effect of SGLT2i drug use, but this has not been reported in horses, and the risk of hypoglycaemia is considered to be low in humans that are not concurrently administered insulin or insulin secretagogues [38]. Other adverse effects of SGLT2i drugs that have been reported to occur in humans include mycotic infections [39,40], genital infections [40], Fournier’s gangrene [41,42,43,44], sarcopenia [45], ketoacidosis [46,47,48,49,50], and bone fractures [51].

The aims of the present study were to determine the pharmacokinetics and safety of one supratherapeutic enteral dose of ertugliflozin in clinically normal adult horses. It was hypothesised that a single supratherapeutic dose of ertugliflozin enterally in clinically normal horses would not be associated with adverse effects, and that ertugliflozin pharmacokinetics in horses would be established, allowing for recommendations for the optimal dose rate and interval.

## 2. Materials and Methods

Horses

The experimental design of this study was approved by the institution’s animal care and ethics committee (A24366 and A24365), and all horses used in the study were provided by the institution’s research herd. For pharmacokinetic analysis, eight clinically normal geldings were used. The number of horses used (*n* = 8) is aligned with similar pharmacokinetic studies and guidelines in animals [30,31,52,53]. No horses had clinical findings or a history of ID, HAL or pituitary pars intermedia dysfunction (PPID). The horses were acclimatised to housing in stables, and underwent physical examinations, oral sugar test (OST), haematological and blood biochemical analyses to confirm suitability as healthy horses. There were seven Thoroughbreds and one Standardbred horse. The median age was 11.5 years (range 3–17), the median body weight was 575 kg (range 484–658), the median body condition score was 5/9 (range 4–6) [54] and the median cresty neck score was 2 (range 1–3) [55]. Demeanour, appetite, frequency of urine output, faecal production, and vital signs (heart rate, respiratory rate, mucous membrane appearance, capillary refill time, rectal temperature and intestinal sounds) were recorded for each horse daily. Horses were allowed ad libitum water access and fed ad libitum lucerne hay and a commercial pellet (Hygain Tru Care^®^, Hygain, Officer VIC, Australia) twice daily while in stables.

### 2.1. Plasma Extraction and Analysis by High-Performance Liquid Chromatography–Tandem Mass Spectrometry

Stored plasma samples were defrosted, and plasma (2 or 4 mL) was pipetted into a 50 mL centrifuge tube and spiked with remogliflozin internal standards. The tube was vortexed, and acetonitrile was added (6 mL for 2 mL plasma, 10 mL for 4 mL plasma). Anhydrous MgSO_4_ (2–3 g) was added, and the tube was capped, vortexed for two minutes, and then centrifuged for five minutes at 4200× *g*. The acetonitrile supernatant was recovered and evaporated to dryness under nitrogen at 30 °C. The residue was resuspended in 250 µL acetonitrile for analysis by high-performance liquid chromatography–tandem mass spectrometry (HPLC-MS).

The column was a Phenomenex Kinetex XB-C18 (50 mm × 3 mm × 1.7 mm) (Phenomenex Australia Pty Ltd., Lane Cove, NSW, Australia) at 40 °C and the injection volume was 5 µL. The mobile phase was 0.05% ammonium sulphate in water (A) and 0.05% ammonium sulphate in 90% acetonitrile (B). The programme held at 90% A for 0.5 min and then changed to 10% A over the next 3.5 min. After holding for two minutes, the original conditions were reestablished. The tandem mass spectrometry (MS/MS) was run in electrospray ionisation (ESI)+ mode and the source conditions were gas temperature (350 °C), gas flow (13 L/min), nebuliser pressure (40 psi), capillary voltage (5000 V), sheath gas temperature (400 °C), sheath gas (12 L/min), and nozzle voltage (2000 V). The ion transitions are shown below (Table 1).

### 2.2. Dosing and Sampling—Oral Sugar Test, Haematological and Blood Biochemical Assessment

**Figure 1:**
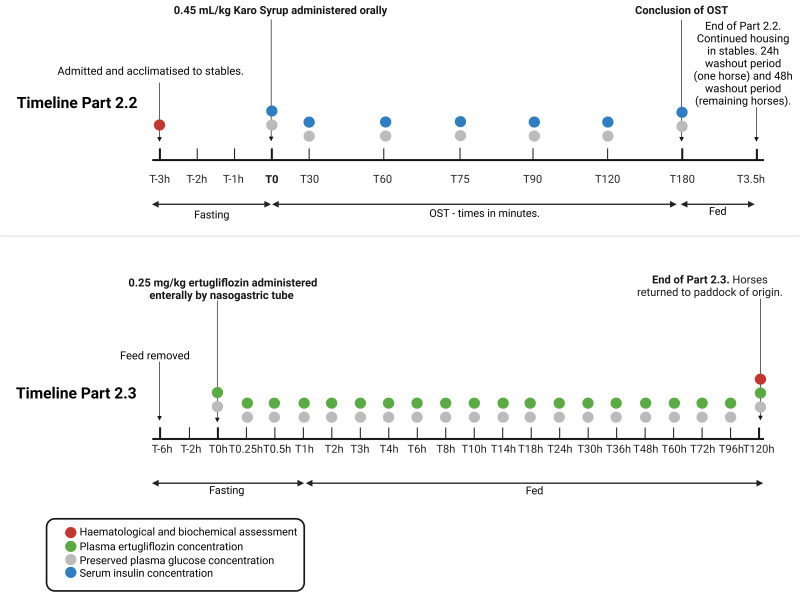
Experimental timeline for parts 2.2 and 2.3. Figure created in BioRender: https://BioRender.com (accessed on 27 April 2026).

All horses were weighed and underwent physical examination prior to relocation from paddocks to individual stalls. A 14-gauge, 5.25-inch catheter (BD Angiocath™, North Ryde, NSW, Australia) was placed in the jugular vein of all horses aseptically upon admission to the stables. The catheters were flushed with heparinised saline regularly to ensure patency. Blood was collected into plain, fluoride/oxalate, sodium citrate and ethylenediaminetetraacetic acid (EDTA) plastic (Vacutainer) tubes (BD Becton, Dickinson and Company, Australia, North Ryde, NSW, Australia) for immediate haematological and blood biochemical assessment (including bile acids and triglycerides) (Figure 1). Each horse subsequently underwent an oral sugar test (OST) as the standard test protocol for assessment for ID prior to drug administration. Specifically, horses were fasted for 3 h, administered 0.45 mL/kg corn syrup orally (Karo Light Corn Syrup, ACH Food Companies Inc., Cordova, TN, USA), and blood samples were collected into plain and fluoride/oxalate plastic (Vacutainer) tubes (BD Australia, North Ryde, NSW, Australia) via the catheter at 0, 30, 60, 75, 90, 120 and 180 min post administration of corn syrup for assessment of insulin and glucose concentrations at each time point (Figure 1). Hyperinsulinaemia was defined by a resting (time 0 min) serum insulin concentration > 45 μIU/mL, and/ or post-prandial (30, 60, 75, 90, 120, 180 min post oral sugar administration) serum insulin concentration >63 μIU/mL [56]. Blood collected into plain tubes was centrifuged at 1.9× *g* and serum was collected, frozen and stored at −20 °C within one hour of collection until the analysis of insulin concentrations. Blood collected into fluoride/oxalate tubes was analysed for glucose concentrations within 3 h of sample collection. Haematological and blood biochemical analyses were performed at a commercial veterinary diagnostic laboratory. One horse underwent a 24 h washout period after the administration of corn syrup, and the remaining horses underwent a 48 h washout period prior to administration of ertugliflozin for pharmacokinetic analysis.

### 2.3. Dosing and Sampling—Pharmacokinetic Analysis

A total of 1300 mg pharmaceutical grade ertugliflozin L-pyroglutamic acid (Azico Biophore, Hyderabad, Telangana, India) was dissolved in 50 mL of dimethyl sulfoxide (DMSO) to ensure complete dissolution (Sigma-Aldrich Pty Ltd., Sydney, NSW, Australia), then mixed with 950 mL water to yield a 1 mg/mL ertugliflozin solution for administration. A 0.25 mg/kg supratherapeutic dose of ertugliflozin was selected to obtain sufficient data points to calculate the elimination half-life (T_1/2_) of ertugliflozin. To accurately estimate the terminal slope of a curve, the sampling times must extend to at least three times the T_1/2_ of a drug [57]. The 0.25 mg/kg dose was also used to establish safety of ertugliflozin, as dose-escalation studies have not been reported in horses.

Given that SGLT proteins are responsible for near 100% fractional glucose reabsorption [18,19], hypoglycaemia was a possible adverse effect of supratherapeutic ertugliflozin administration in healthy horses. One horse underwent drug administration 24 h prior to the remaining horses to determine if hypoglycaemia occurred and mitigate risks of adverse effects in other horses.

Blood samples for pharmacokinetic analysis were collected over a 5-day period in a deescalating pattern. Specifically, the horses were subjected to a 6 h period of fasting, and blood samples were collected via the catheter before (time 0) and 0.25, 0.5, 0.75, 1, 2, 3, 4, 6, 8, 10, 14, 18, 24, 30, 36, 48, 60, 72, 96, and 120 h after the administration of 0.25 mg/kg of pre-prepared 1 mg/mL ertugliflozin solution by nasogastric tube, immediately followed by 1 L water (Figure 1). Blood was collected via the catheter into a syringe and transferred within 30 s into fluoride/oxalate and heparin-coated plastic tubes for the assessment of glucose and ertugliflozin concentrations, respectively. Blood collected in heparin-coated tubes was centrifuged at 1.9× *g* and heparinised plasma was separated and frozen at −20 °C within one hour of sample collection until analysis. Whole blood from the syringe was used to measure the glucose concentration immediately after collection using a glucometer, and the blood collected in fluoride/oxalate tubes was submitted for laboratory determination of blood glucose concentration within 3 h of sample collection. At the 120 h sampling point, blood was collected into plain, sodium-citrate, fluoride/oxalate and EDTA tubes for haematological and blood biochemical analysis (including triglycerides and bile acids) to investigate the safety profile of the drug. Following the 120 h sample, the catheters were removed, and the horses were returned to the paddock of origin (Figure 1). A control group was not required for the study design, as the pharmacokinetic variables were determined by drug administration only.

### 2.4. Pharmacokinetic and Safety Analysis

The blood biochemical analytes were measured using an automatic biochemistry analyser (Beckman Coulter AU480 biochemistry analyser, Beckman Coulter, California, USA). The insulin concentrations were measured by a chemiluminescent immunoassay (Siemens IMMULITE^®^ 1000, Siemens Healthcare Diagnostics, Erlangen, Germany) validated for use in horses [58]. The blood glucose concentrations were measured using point-of-care glucometry with whole blood (Accu-Chek^®^, Roche Diagnostics Corp, Indianapolis, IN, USA), and from fluoride/oxalate tubes using an automatic biochemistry analyser (Beckman Coulter AU480 biochemistry analyser) within 3h of blood collection. Two glucose concentration measurements were obtained for determination of the precision of the Accu-Chek^®^ and monitoring for hypoglycaemia.

Plasma concentrations of ertugliflozin were determined by HPLC-MS. The maximum measured concentration (C_max_) and time to maximum concentration (T_max_) of ertugliflozin were determined directly from the data. Other pharmacokinetic variables from the ertugliflozin concentration vs. time data were determined for each horse by use of noncompartmental analysis with PKSolver 2.0 software.

The area under the concentration time curve from zero to infinity (AUC0–∞) and area under the first moment curve (AUMC0–∞) were calculated by use of the linear trapezoidal rule. The mean residence time (MRT0–∞) was calculated as AUMC0–∞/AUC0–∞, where AUMC0–∞ is the area under the first moment curve from 0 to infinity. The terminal elimination half-life (T_1/2_) was calculated as (ln 2)/λz, where λz is the terminal elimination rate constant calculated by means of log-linear regression. Calculation of T_1/2_ involved the use of the last six data points for each horse. The apparent oral clearance was calculated as CL/F.

### 2.5. Statistical Analysis

The data were collated in Microsoft Excel (Microsoft, Sydney, Australia). No data were excluded from analysis. The data were assessed for normality using the Shapiro–Wilk test, and the estimates of central tendency and spread of data for haematological and blood biochemical variables were described: mean ± standard deviation for normally distributed data and the median and interquartile range for non-normally distributed data.

## 3. Results

**Figure 2:**
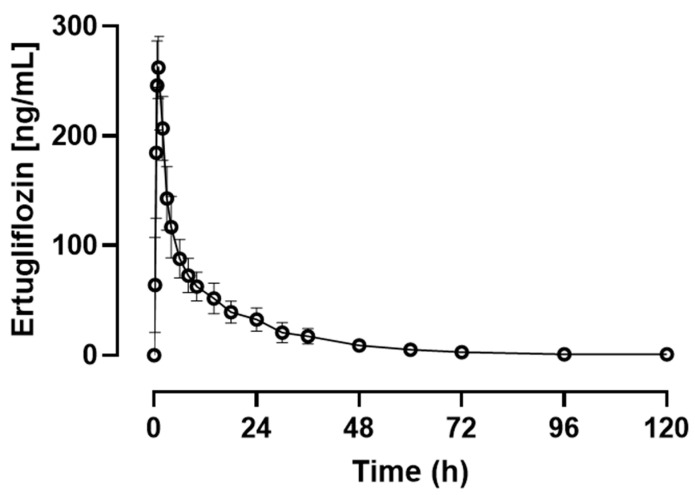
Mean ± SD plasma concentrations of ertugliflozin after enteral administration of ertugliflozin to 8 adult horses at a dose of 0.25 mg/kg. Figure created in GraphPad Prism (10.0.3).

For all eight horses, ertugliflozin concentrations were quantified from 0.25 h to 120 h (Figure 2, Supplementary Table S1). Plasma concentrations at time 0 h were below the limit of detection. Mean ± SD T_1/2_ and CL/F of ertugliflozin were 17.65 ± 3.15 h and 106.95 ± 27.53 mL/h/kg, respectively. The pharmacokinetic data for ertugliflozin are summarised in Table 2.

**Figure 3:**
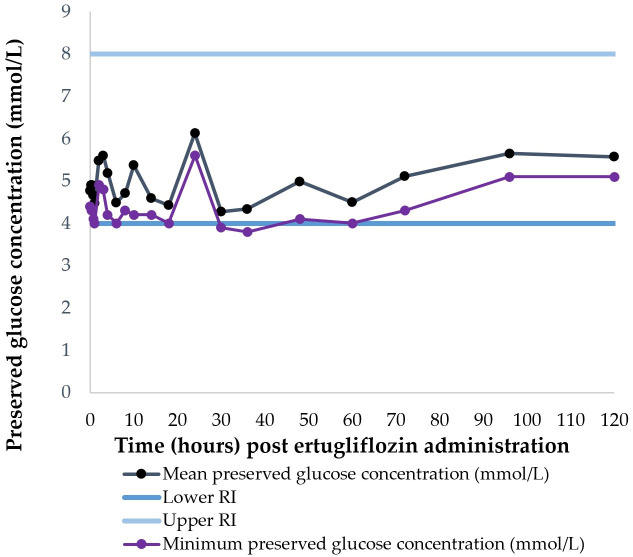
Mean and minimum preserved plasma glucose concentrations assessed at a commercial laboratory in horses after receiving a supratherapeutic dose of ertugliflozin enterally at T0. Horses were fasted for 6 h prior to drug administration and were fed at T1 h. Horses received ad libitum access to feed after T1 h.

No clinical signs of adverse effects were observed. Daily physical examination revealed vital signs within normal limits throughout the study period. Mean ± SD glucose concentration throughout the study period was 5.0 ± 0.5 mmol/L. All horses were normoglycemic for the first 24 h after ertugliflozin administration. Three horses developed mild hypoglycaemia (preserved samples analysed at a commercial veterinary diagnostic laboratory) (3.8–3.9 mmol/L, reference interval (RI) 4–8 mmol/L) between 30 and 36 h after drug administration that was resolved without intervention. However, point-of-care glucometry on the same sample did not reveal hypoglycaemia. The mean and minimum preserved glucose concentrations for all horses that were determined by a commercial laboratory during the study period are shown in Figure 3.

All horses in the study demonstrated normal insulin responses to oral sugar administration: 0, 30, 60, 75, 90, 120 and 180 min after administration of 0.45 mg/kg corn syrup orally (Karo Light Corn Syrup, ACH Food Companies Inc, Cordova, TN, USA).

No haematological or blood biochemical abnormalities occurred after drug administration. The summary of the haematological and blood biochemical data before and five days after a single supratherapeutic ertugliflozin administration can be found in Supplementary Table S2.

## 4. Discussion

Determination of the pharmacological profile of ertugliflozin in horses is required for evidence-based use of this drug. Despite the widespread clinical use of ertugliflozin in insulin-dysregulated horses, dosing regimens have been based on anecdotal experiences rather than pharmacokinetic–pharmacodynamic integration. In the present study, the key pharmacokinetic variables (T_1/2_ and CL/F) of ertugliflozin were established after a single supratherapeutic dose enterally, allowing recommendations to be made for the dose rate and interval. One supratherapeutic administration of ertugliflozin was well tolerated and there were no observed adverse effects in the horses.

The bioavailability and actual clearance of ertugliflozin in the horses cannot be reported as intravenous administration is required to determine these pharmacokinetic features of the drug. In humans, the oral bioavailability of ertugliflozin was estimated at 100% using a novel micro-dose [14C] radio-labelled ertugliflozin method [59]. Without determination of pharmacokinetics of ertugliflozin after intravenous administration in the horse, the actual clearance and bioavailability remain unknown and might differ from the apparent values. Research in humans suggests that ertugliflozin can be administered in the fed or fasted state [60]. The impact of feeding and fasting on the pharmacokinetics of enterally administered ertugliflozin were not investigated in the present study. Ertugliflozin was administered in the fasted state to minimise the likely effects of high-fibre feed on oral absorption [61].

In the present study, the ertugliflozin was prepared with DMSO to ensure complete drug dissolution, as the drug has poor water solubility. Complete dissolution in a liquid carrier was important to confirm administration of the entire drug dose, for accurate pharmacokinetic assessment. In horses that undergo oral administration (e.g., tablet or oral preparation), there is a risk of drug loss or wastage due to poor compliance, which is inappropriate for pharmacokinetic studies. Therefore, the 5% DMSO and ertugliflozin solution was administered enterally by nasogastric tube, rather than orally. At a 5% DMSO concentration immediately followed by 1L water (~0.5% DMSO concentration after dilution), it is unlikely that the addition of DMSO altered the pharmacokinetic variables of ertugliflozin. However, DMSO is not a carrier of the drug used in clinical practice.

The dose of ertugliflozin used for pharmacokinetic assessment was 0.25 mg/kg and was selected rather than the 0.02–0.06 mg/kg (0.05 mg/kg most commonly) dose range reported for use in horses [26,28,56] based on dose escalation studies in humans that have shown that ertugliflozin exhibits linear pharmacokinetics and safety within the range of 0.05–4 mg/kg [62,63], and to obtain sufficient data points to calculate T_1/2_ [57].

The mean elimination half-life and apparent oral clearance in horses and humans are comparable. In the present study, the T_1/2_ was 17.65 ± 3.15 h and CL/F was 106.95 ± 27.53 mL/h/kg versus a T_1/2_ of 10–17 h [64] and CL/F of approximately 150 mL/h/kg reported in human pharmacokinetic studies [65]. This finding is unexpected because drugs that are metabolised in the liver are, in general, absorbed less efficiently and cleared faster in horses than in humans [66]. The 17 h half-life makes ertugliflozin suitable for once-daily dosing in the horse, with the steady state predicted to be reached within 4 days (4–5 × T_1/2Z_).

The currently recommended dose range of 0.02–0.06 mg/kg reported for use in horses [26,28,56] was evaluated based on our pharmacokinetic data. In a meta-analysis of 25 phase I pharmacokinetic studies in humans, ertugliflozin was found to exhibit linear pharmacokinetics, with AUC and C_max_ dose-proportionality being within the range of 0.05–4 mg/kg [64]. Pharmacodynamic–pharmacokinetic integration modelling was used in the development and approval (US and EU) of ertugliflozin in people, with maximal urinary glucose excretion being reached at 0.2 mg/kg; the current approved dose range in humans is approximately 0.07–0.2 mg/kg (5 mg or 15 mg tablet per person once daily) [64]. The AUC of ertugliflozin in humans, dosed at 0.2 mg/kg, is approximately 1500 ng·h/mL [56,57]. The AUC of ertugliflozin given at 0.25 mg/kg to the horses in the present study was 2473.90 ± 626.72 ng·h/mL. If dose proportionality occurs in horses, as in humans, the estimated AUC of ertugliflozin dosed at 0.05 mg/kg would be 494.78 ng·h/mL. Considering that the pharmacokinetics of ertugliflozin in horses are comparable to humans, and the current approved dose range in humans is 0.07–0.2 mg/kg, it is probable that the 0.05 mg/kg ertugliflozin dose used in horses is at the lower end of the equine therapeutic range.

There was no clinical or laboratory evidence of adverse effects of a single supratherapeutic administration of ertugliflozin to horses in the current study. A significant increase in the triglyceride concentration and activity of glutamate dehydrogenase (GLDH) after single supratherapeutic administration of canagliflozin in healthy horses [30], as well as increased activity of gamma glutamyl transferase (GGT) in horses with ID treated with ertugliflozin or dapagliflozin [28], have previously been reported; this was not a feature in the present single-dose study. Further multiple dose-regimen administration studies in diseased horses are required to determine long-term safety of ertugliflozin. Three horses developed mild, sub-clinical hypoglycaemia assessed on preserved samples analysed at a commercial veterinary diagnostic laboratory that resolved without intervention and were likely biologically irrelevant (Figure 3). Hypoglycaemia secondary to SGLT2i administration has not previously been reported in horses, and the risk of hypoglycaemia is considered to be low in humans that are not concurrently administered insulin or insulin secretagogues [38]. The low risk of hypoglycaemia might be due to increased endogenous glucose production [67,68], decreased renal threshold for glucose excretion [38] or increased SGLT1-mediated transport [17].

In this study, light-breed adult geldings that did not have insulin dysregulation were used. These animals were selected given the minimal diversity in breed, body condition and body weight, as well as research herd availability. A population of healthy animals with comparable pharmacokinetics is typical for pharmacokinetic studies in animals and humans before investigating pharmacokinetics in diseased animals. A key direction for future research is to determine whether horses with hyperinsulinaemia demonstrate similar pharmacokinetics to healthy horses after oral administration of ertugliflozin. Physiologically based pharmacokinetic modelling and determining whether there are sex or breed differences in pharmacokinetics are also areas that require further investigation.

The authors acknowledge the limitations of the present study. The pharmacokinetics of ertugliflozin in horses with ID was not established and remains a key direction for future research. Ertugliflozin was administered enterally, rather than orally, and prepared with DMSO to ensure complete drug dissolution. This is unlikely to have altered the pharmacokinetics. However, the pharmacokinetic variables might differ slightly when compared to orally administered, commercial drug preparations. Horses were not definitively excluded for PPID prior to inclusion in the study population. Thyrotropin releasing hormone (TRH)-stimulated ACTH concentrations could have been assessed in horses >10 years of age. However, a definitive diagnosis could not have been made based on ACTH concentrations alone in this population, due to low pre-test probability [69] and the risk of false positive test results. Additionally, the pharmacokinetics of ertugliflozin were only assessed in large-breed geldings; no ponies, mares or stallions were included in the study population.

One supratherapeutic administration of ertugliflozin is well tolerated with no observed adverse effects in healthy horses. Considering that the pharmacokinetics of ertugliflozin in horses are comparable to humans, and the current approved dose range in humans is 0.07–0.2 mg/kg, it is probable that the 0.05 mg/kg ertugliflozin dose used in horses is at the lower end of the therapeutic range. It is proposed that a starting dose for ertugliflozin in horses be extended to the range of 0.05–0.1 mg/kg. Further studies are warranted to determine the ertugliflozin dose for optimal efficacy in the treatment of ID horses, thereby optimising a dose range for individualisation of therapy.

## Figures and Tables

**Table 1:** Ion transitions during MS/MS run in ESI+ mode.

	Precursor	Product	Fragmentor (V)	Collision (V)	Capillary Voltage (V)	Dwell (ms)
Remogliflozin (internal standard)	451.2	289.2	120	13	4	20
Ertugliflozin (quantifier)	437.0	329.0	120	7	5	100
Ertugliflozin (qualifier)	437.0	207.0	120	18	5	20

**Table 2:** Pharmacokinetic variables of ertugliflozin in horses following a single enteral dose of 0.25 mg/kg. Results are expressed as mean ± SD, *n* = 8.

Variable (Units)	Mean ± SD
C_max_ (ng/mL)	267.52 ± 25.37
T_max_ (h)	0.91 ± 0.13
λz (h^–1^)	0.041 ± 0.009
T_1/2Z_ (h)	17.65 ± 3.15
AUC_0–t_ (ng·h/mL)	2453.00 ± 614.60
AUC_0–∞_ (ng·h/mL)	2473.90 ± 626.72
MRT_0–∞_ (h)	17.72 ± 2.61
CL/F (mL/h/kg)	106.95 ± 27.53

C_max_ = maximum concentration. T_max_ = time to maximum concentration. λz = terminal elimination rate constant. AUC_0–t_ = area under the curve from 0 to last quantifiable time point. AUC0–∞ = area under the curve from 0 to infinity. MRT0–∞ = mean residence time extrapolated to infinity. CL/F = apparent oral clearance.

## Data Availability

The original contributions presented in this study are included in the article/Supplementary Materials. Further inquiries can be directed to the corresponding author.

## Supplementary Materials

The following supporting information can be downloaded at: https://www.mdpi.com/article/10.3390/vetsci13050445/s1. Table S1: Mean ± SD plasma concentrations of ertugliflozin after enteral administration to 8 adult horses at a dose of 0.25 mg/kg; Table S2: Summary data, haematologic and blood biochemical analyses before and five days after a single administration of a supratherapeutic dose of ertugliflozin enterally. The estimates of central tendency and spread of data are provided based on data distribution.
