# Supplementary material for: Pharmacokinetics of Ertugliflozin, a Sodium-Glucose Co-Transporter-2 Inhibitor (SGLT2i) in Horses After Enteral Administration
Source: Vet Sci. 2026 May 1;13(5):445. doi: 10.3390/vetsci13050445 (PMC13211585; doi:10.3390/vetsci13050445)
Supplement: Supplementary file 1 [file vetsci-13-00445-s001.zip › vetsci-4233428-supplementary.pdf]

Pharmacokinetics of Ertugliflozin, a Sodium-Glucose Co-Transporter-2 Inhibitor (SGLT2i) in Horses After Enteral Administration – Supplementary tables and figures.

Supplementary Table S1: Mean ± SD plasma concentrations of ertugliflozin after enteral administration to 8 adult horses at a dose of 0.25 mg/kg.

Time (h)	Mean [ertugliflozin] (ng/mL)	SD (ng/mL)
0	0	0
0.25	64.05	43.27
0.5	184.71	59.76
0.75	245.89	40.66
1	262.49	28.43
2	206.83	29.23
3	142.86	28.89
4	116.87	28.04
6	87.89	17.42
8	72.64	15.50
10	62.55	13.23
14	51.80	13.81
18	39.27	9.91
24	32.54	10.65
30	20.59	9.39
36	17.36	6.98
48	9.03	4.76
60	5.23	2.79
72	2.87	1.67
96	0.91	0.53
120	0.87	0.54

Supplementary Table S2: Summary data, haematologic and blood biochemical analyses before and five days after a single administration of a supratherapeutic dose of ertugliflozin enterally. The estimates of central tendency and spread of data are provided based on data distribution.

Analyte	Reference range		Mean	SD	Median	IQR
Red Blood Cell Count	6.5-12.5 x 10^{12}/L	Pre	8.9 x 10^{12}	0.9		
		Post	7.9 x 10^{12}	0.7		
Haemoglobin	110 – 190 g/L	Pre	144.0	15.3		

		Post	126.9	12.5		
Haematocrit	0.32 – 0.52 L/L	Pre	0.41	0.04		
		Post	0.37	0.04		
MCV	34 – 58 fL	Pre	46.6	2.1		
		Post	46.9	2,6		
MCH	12 – 18 pg	Pre	16.3	0.7		
		Post	16.3	0.7		
MCHC	310 – 370 g/L	Pre	348.4	4.2		
		Post			349.5	7.5
Platelets	100 – 500 x10^9/L	Pre	142.5 x10^9	18.3 x10^9		
		Post	126.4 x10^9	22.4 x10^9		
White Blood Cell Count	5.5 – 12.5 10^9/L	Pre	7.7 x10^9	1.7 x10^9		
		Post	6.0 x10^9	1.0 x10^9		
Neutrophils	2.5 – 8.0 10^9/L	Pre			4.4 x10^9	1.7 x10^9
		Post	3.5 x10^9	0.8 x10^9		
Lymphocytes	1.5 – 5.5 10^9/L	Pre	3.1 x10^9	0.6 x10^9		
		Post	2.2 x10^9	0.5 x10^9		
Monocytes	0.0 – 0.9 10^9/L	Pre	0.2 x10^9	0.2 x10^9		
		Post			0.2 x10^9	0.1 x10^9
Eosinophils	0.0 – 0.8 10^9/L	Pre	0.3 x10^9	0.2 x10^9		
		Post	0.1 x10^9	0.1 x10^9		
Basophils	0.0 – 0.3 10^9/L	Pre			0.0 x10^9	0.1 x10^9
		Post			0.0 x10^9	0.1 x10^9
Total Protein	53 – 75 g/L	Pre	62.9	3.0		
		Post	62.3	3.3		
Albumin	27 – 39 g/L	Pre	33.0	2.1		
		Post	34.3	2.2		
Globulins	20 – 40 g/L	Pre	30.0	4.2		

		Post	28.0	4.0		
Creatinine	81 – 164 µmol/L	Pre	97.9	6.2		
		Post	111.0	6.9		
Urea	3.2 – 8.1 mmol/L	Pre	7.8	0.7		
		Post	7.2	0.4		
CK	113 – 375 U/L	Pre	466.9	199.2		
		Post	248.8	67.4		
AST	194 – 440 U/L	Pre			324.5	6.8
		Post	327.8	35.9		
GLDH	0 – 20 U/L	Pre	5.5	1.9		
		Post	3.5	1.4		
ALP	1 – 280 U/L	Pre			139.5	65.8
		Post	193.6	77.1		
Bilirubin	15 – 75 µmol/L	Pre	18.8	4.1		
		Post	30.4	6.8		
GGT	9 – 40 U/L	Pre			14.0	4.0
		Post			14.0	1.0
Triglycerides	< 0.9 mmol/L	Pre	0.3	0.1		
		Post	0.2	0.1		
Potassium	3.0 – 5.0 mmol/L	Pre	3.9	0.2		
		Post	3.5	0.4		
Sodium	133 – 143 mmol/L	Pre	136.8	1.3		
		Post			136.0	4.0
Anion Gap	6 – 18 mmol/L	Pre	9.9	0.99		
		Post	11.8	1.39		
Calcium	2.7 – 3.3 mmol/L	Pre	2.9	0.1		
		Post	2.9	0.1		
Phosphate	0.8 – 1.8 mmol/L	Pre	1.1	0.2		

		Post	1.2	0.2		
Chloride	96 – 104 mmol/L	Pre	100.1	1.5		
		Post	99.1	1.7		
Bicarbonate	23 – 32 mmol/L	Pre	30.8	1.4		
		Post			29.5	2.0
Magnesium	0.6 – 1.0 mmol/L	Pre	0.7	0.0		
		Post			0.6	0.1
Bile Acids	0.0 – 15.0 μmol/L	Pre	4.0	1.3		
		Post	8.1	2.9		
Fibrinogen	1.0 – 4.0 g/L	Pre	1.6	0.2		
		Post	1.7	0.2		
